# Feasibility of a pilot dyadic randomized controlled trial testing the effects of three behavioral interventions on older adults’ cognitive, physical and everyday function

**DOI:** 10.3389/fragi.2023.1166338

**Published:** 2023-05-25

**Authors:** Christine B. Phillips, Ava McVey, Junyan Tian, Abigail T. Stephan, W. Bennett Davis, Erica L. Aflagah, Lesley A. Ross

**Affiliations:** ^1^ Department of Psychology, Clemson University, Clemson, SC, United States; ^2^ Clemson University Institute for Engaged Aging, Seneca, SC, United States; ^3^ Human Development and Family Studies, Pennsylvania State University, University Park, PA, United States; ^4^ Department of Public Health Sciences, Clemson University, Clemson, SC, United States; ^5^ Department of Neurology, Neuropsychology Division, University of Nebraska Medical Center, Omaha, NE, United States

**Keywords:** exercise, training, exergames, physical, cognitive, everyday function, behavioral interventions

## Abstract

**Introduction:** Maintaining functional abilities is critical for optimizing older adults’ well-being and independence. This randomized controlled trial (RCT) pilot examined the feasibility of testing the effects of three commercially available interventions on function-related outcomes in older adults.

**Methods:** Pairs of community-dwelling older adults (N=55, Mage=71.4) were randomized to a 10-week intervention (cognitive-COG, physical-EX, combined exergame-EXCOG, or control-CON). Cognitive, physical, and everyday function were assessed at baseline, immediately post-intervention, and 6-months post-intervention. Feasibility was evaluated using recruitment, enrollment, training adherence, and retention metrics. Variability and patterns of change in functional outcomes were examined descriptively.

**Results:** A total of 208 individuals were screened, with 26% subsequently randomized. Across training arms, 95% of training sessions were completed and 89% of participants were retained at immediate post-test. Variability in functional outcomes and patterns of change differed across study arms.

**Discussion:** Results support a fully powered RCT, with several modifications to the pilot study design, to investigate short- and long-term training impacts.

## 1 Introduction

Living independently is predicated upon the maintenance of cognitive, physical, and everyday functions required to successfully perform instrumental activities of daily living (IADL). Many complex IADL, also called everyday functions, require both cognitive (e.g., attention, speed of processing, memory) and physical (e.g., balance, mobility) abilities (Gold, 2012; BrudererHofstetter et al., 2020). These activities often include meal planning and preparation, transportation, and managing health affairs and finances. Continued successful participation in these activities in older adulthood is a marker of healthy aging (Katz, 1983), and ensuring older adults maintain cognitive, physical, and everyday function is critical for optimizing their wellbeing and independence.

The current Cognitive and Physical Exercise Study (CAPES) pilot explores the feasibility of conducting a dyadic randomized controlled trial testing three commercially available training interventions. The intervention types deployed were a) computerized cognitive training, b) physical exercise, and c) simultaneous combined cognitive and physical exercise (i.e., exergames). We chose a dyadic randomization approach based on previous research suggesting that peer/friend dyads and/or having shared goals can promote training adherence among older adults (Carr et al., 2019). The following sections provide an overview of each training type, study methods, and results evaluating feasibility indicators and variability and patterns of change in functional outcomes by study arm. A discussion with conclusions and recommendations for a future larger scale trial is provided.

Cognitive training interventions incorporate activities designed to improve brain function across a range of cognitive domains, such as processing speed, attention, memory, and executive function (Simons et al., 2016; Butler et al., 2018). Multiple review papers have found that cognitive training interventions improve cognitive performance in specific domains of training in older adults (i.e., near transfer effects; Butler et al., 2018; Ten Brinke et al., 2020; Willis et al., 2006), though results are mixed regarding whether benefits transfer to tasks or cognitive domains other than those trained (i.e., far training transfer effects; Sala et al., 2019; Tetlow and Edwards, 2017; Zelinski, 2009). Cognitive training interventions, especially those tapping process-based constructs such as processing speed and attention, have shown transfer to improved physical function outcomes such as gait and balance among healthy, community-dwelling older adults (e.g., Verghese et al., 2010; Smith-Ray et al., 2014; SmithRay et al., 2015; Azadian et al., 2018; Marusic et al., 2018; Ross et al., 2018; Sprague et al., 2019). Additionally, physical function benefits conferred from cognitive training may persist for as long as a decade post-intervention (Ross et al., 2018; Sprague et al., 2019). Similarly, process-based cognitive training has been shown to improve objectively-assessed everyday function (Ball et al., 2007; Chen et al., 2018; Edwards et al., 2018), including driving mobility (Ross et al., 2016; 2017) and safety (Roenker et al., 2003; Ball et al., 2010) between immediate post-test up to 10- years.

An abundance of research has shown that physical exercise, particularly multi-component interventions targeting strength, endurance, and balance, decrease fall incidence and frailty and increase muscle strength and overall physical functioning (e.g., Cadore et al., 2013; Dipietro et al., 2019). The effects of physical exercise interventions on cognitive function are less conclusive; however, results have generally indicated that physical exercise interventions can improve cognitive abilities, such as executive functioning, spatial and speed processing, working memory, and memory (Colcombe and Kramer, 2003; Sanders et al., 2019; Zhidong et al., 2021). To our knowledge only McDaniel et al. (2014) have investigated physical exercise for improving the ability to perform cognitively complex everyday activities. These authors found aerobic exercise training did not improve simulated IADL task performance.

Combined interventions, including both cognitive and physical exercise training, may produce synergistic benefits relevant to older adults’ functional abilities that are larger than each intervention on its own (Bherer et al., 2021). As an extension of combined interventions, those that incorporate game elements (i.e., exergames) have gained popularity because of their interactive and motivational attributes (Chao et al., 2015a; Nawaz et al., 2016). Numerous studies have shown exergames improve multiple aspects of physical functioning, including balance, lower extremity strength and postural control among older adults (Chao et al., 2015b; BrudererHofstetter et al., 2018; Kappen et al., 2018). In contrast, fewer studies have investigated the effects of exergaming on cognitive and everyday functional outcomes, with varied results. While some evidence suggests exergaming interventions improve cognitive and dual-task (i.e., combined physical and cognitive) functioning (BrudererHofstetter et al., 2018; Ogawa et al., 2019; Gallou-Guyot et al., 2020; Jiang et al., 2022), other findings indicate little to no benefit of exergames across a range of cognitive outcomes (Ordnung et al., 2017; Sala et al., 2021). No studies to our knowledge have examined the effects of exergaming interventions on cognitively complex IADL among healthy older adults. Moreover, there is a lack of research comparing commercially available interventions. Many gamified and cognitive interventions are experiment-specific, challenging the generalizability, reproducibility, and translation of findings (Lumsden et al., 2016). Additionally, this raises accessibility and practical limitations of findings in the current literature.

Cognitive, physical, and combined interventions may preserve functional abilities in older adults. However, optimal protocols and differential impact of training type on functional abilities have not been extensively investigated. The primary aim of this 4-arm, single-blind, dyadic randomized intervention pilot study was to examine the feasibility of conducting a fully powered trial testing the effects of three commercially available interventions on multiple function-related outcomes in older adults. To address this aim, we assessed length of time required to complete participant recruitment, ratio of screened to randomized individuals, intervention adherence and retention rates, and missing data. A second aim was to estimate the variability in executive, useful field of view (UFOV), physical and everyday function, by generating interval estimates of the mean difference from baseline to immediate post-test and 6-month follow-up for each outcome measure. Lastly, we aimed to explore patterns of functional changes by study arm by graphically visualizing mean functional performance over time (i.e., each assessment point). Taken together, we anticipate the results will inform the design of a subsequent larger-scale trial to test and directly compare the effectiveness of multiple behavioral interventions for improving or preserving function among older adults.

## 2 Materials and methods

### 2.1 Study design

**FIGURE 1:**
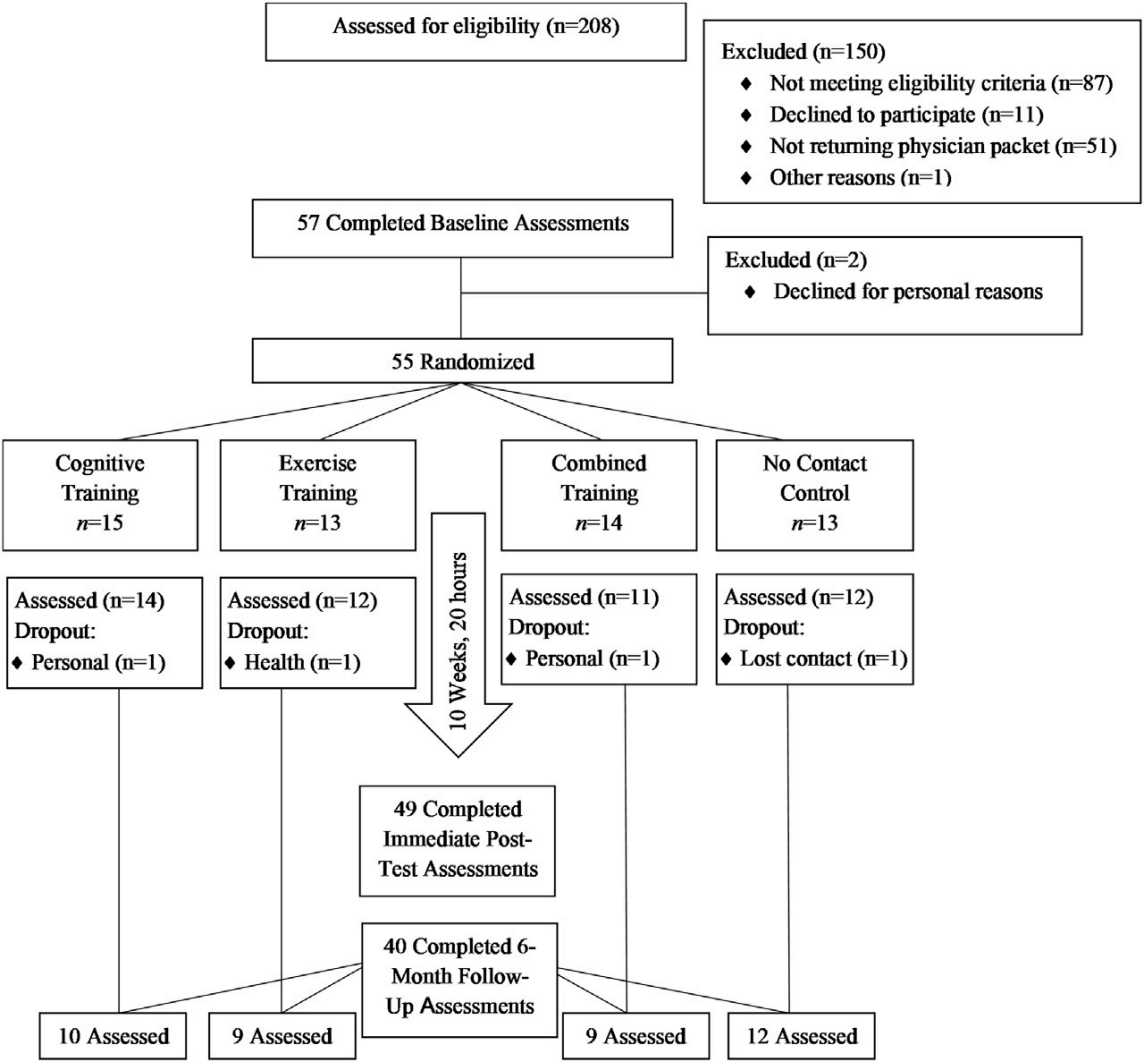
Consort flow diagram.

The Cognitive and Physical Exercise Study (CAPES) (OSF registration, https://osf.io/ka5gy/?view_only=69968ec81d7246d6abb80 ce887099a8d) was a 10-week, 4-arm, single-blind, randomized feasibility trial testing the effects of three commercially available interventions *versus* a no-contact control on multiple functional outcomes. Following baseline assessment, older adults (N = 55) were randomly assigned in dyads to one of four conditions with equal allocation. All study assessment and intervention visits took place on a university campus. Eligibility criteria and outcomes were assessed by blinded research personnel among all participants at baseline, immediate post-test and at follow-up visits. Due to funding limitations, only a subsample of individuals (n = 40) randomly selected from each condition were invited for a follow-up assessment 6 months after immediate post-test. Please see Figure 1 for CONSORT flow diagram.

### 2.2 Participants

Healthy older adults residing in the Birmingham, AL metropolitan area were recruited via mailings and posted flyers in 2013. All study visits occurred between April, 2013 and January, 2015. Experimental procedures were approved and conducted within the guidelines set forth by the University of Alabama at Birmingham Institutional Review Board for Human Use. All participants provided written consent prior to enrollment and were compensated for participation in the study.

Inclusion criteria for enrollment were as follows: 1) aged 65–95 years, 2) no more than 2 hours per week of self-reported moderate-to-vigorous physical activity, cognitive training (e.g., Posit, Lumosity), or video games in the previous 2 years, 3) no history of major health conditions known to affect cognitive or physical functioning (i.e., heart problems or disease, stroke, or transient ischemic attack, substance abuse), and 4) no evidence of dementia as assessed by a score >21 on the Modified Telephone Interview for Cognitive Status (TICS-M; Welsh et al., 1993), or self-reported or physician-diagnosed dementia. Eligible participants provided written approval from their physicians attesting to their fitness to participate in the study and could not have exhibited abnormal physiological responses during a baseline in-lab graded exercise test. A total of 57 older adults attended a baseline screening visit. Two participants dropped out prior to randomization, resulting in a randomized sample of 55 older adults. Participants received up to $350 depending on the portions of the study they completed. Participants completing all assessment visits received $150. Those randomized to training received an additional $200 for completing 20 h (sessions). Those who did not finish the study were provided remuneration for the portions they completed. All participant payments were provided within approximately 14 days of the last day of study participation.

### 2.3 Intervention procedure

Participants were paired with a partner of their choosing (typically a spouse or friend)—if both individuals met inclusion criteria, had completed all baseline measurements, and had similar availability for training schedules. For individuals who did not enroll with a partner, a participant of the same sex and approximate age, with similar training availability, was assigned. Using the sealed envelope method, each dyad was randomly assigned by the study coordinator to cognitive training (COG; n = 15), exercise training (EX; n = 13), combined training using exergames (EXCOG; n = 14), or a no-contact control (CON; n = 13). In each training arm, participants attended two, 1-hour sessions per week over a 10-week period (i.e., 20 training sessions) between June, 2013 and September, 2014. Participants in all study arms were informed that the purpose of the study was to investigate “different brain and exercise programs designed to improve brain and physical health.” All three training interventions took place in a social group setting, with the same pair of participants taking part in each session together. If a participant’s partner dropped out prior to completion of at least 2 weeks of training, a new partner was assigned; otherwise, the participant completed the remainder of the training without a partner. Each intervention consisted of three different activities, which were administered in a mixed order to reduce boredom.

#### 2.3.1 Cognitive training (COG)

Participants played three computer-based games using InSight, a trademarked program from Posit Science Corporation (Delahunt et al., 2009). The training games included 10 hours of Road Tour and 5 hours of Bird Safari and Jewel Diver, which were designed to improve individuals’ processing speed, divided attention, and selective attention.

#### 2.3.2 Physical training (EX)

Participants engaged in three different workouts using the Older and Wiser Workout DVD series (Grant, 2010). Videos included Older and Wiser, Older and Much Wiser, and Fit at Any Age. These workouts, designed for older adults, consisted of low-impact cardio aerobics, gentle strength training, balance exercises, and stretching.

#### 2.3.3 Combined exergame training (EXCOG)

Participants played three different sets of games using a commercially available platform, the Xbox 360 Kinect (Microsoft Corporation; Redmond, WA). The games were Just Dance 4, Body and Brain Connection, and Kinect Adventures, and all of them involved some combination of physical and cognitive activities.

### 2.4 Measures

#### 2.4.1 Screening measures

##### 2.4.1.1 Cognitive status

The Modified Telephone Interview for Cognitive Status (TICS-M) was used as a dementia screening assessment (Welsh et al., 1993). Participants responded to questions that evaluated basic cognitive functions including orientation, attention and calculation, memory, comprehension, and language. In accordance with established scoring procedures and cut points indicating possible impairment, participants needed to score >21 to be included in this study.

##### 2.4.1.2 Physical status

A graded exercise test (GXT) was administered and supervised by a certified physiologist at baseline to assess cardiorespiratory function. This test involved supervised walking or running on a treadmill while the pace and incline of the walk or run gradually increased. Participants continued until voluntary exhaustion or test administrators observed at least two of three physiological criteria: an age-adjusted maximal heart rate (i.e., 220 bpm minus age), a respiratory exchange ratio (RER: VCO2 metabolism divided by VO2 usage) greater than 1.15, and a leveling-off of O2 consumption with increasing workload. Throughout the test, participants wore sensors that monitored their heart rate (12-lead electrocardiogram), blood pressure, and oxygen uptake (VO2 max). Participants were deemed ineligible for study participation if any abnormal physiological responses were recorded during the assessment.

#### 2.4.2 Outcome measures

##### 2.4.2.1 Executive function

Based on results from an exploratory factor analysis, a composite variable was created from six measures assessing multiple executive functions, including planning and behavioral organization, problem solving, attentional allocation, cognitive flexibility, abstraction, impulse control, episodic and working memory, psychomotor processing speed, and non-verbal reasoning. Measures included: 1) Neuropsychological Assessment Battery (NAB) Mazes Test (Stern and White, 2009), 2) Trail Making Test Part B (Bowie and Harvey, 2006) 3) Rey Complex Figure Test Copy Trial (Meyers and Meyers, 1995), 4) Wechsler Adult Intelligence Scale, Matrix Reasoning and Block Design subtests (WAIS-IV; Wechsler, 2008), and 6) Tower of London-Drexel University: 2nd Edition (Culbertson and Zillmer, 2005). Where appropriate, scores were reverse coded so that higher scores indicated better performance. Scores for each test were standardized to the baseline mean (i.e., z-scored), then summed and averaged to obtain a composite score at each timepoint.

##### 2.4.2.2 Useful field of view (UFOV)

A set of 4 computerized subtests with speed-of-processing and attention (divided and selective) elements were used to assess participants’ UFOV (Edwards et al., 2006; 2005). All four subtests involved identifying a central object. Each subsequent test built on the previous one by adding simultaneous location of a peripheral object (Subtest 2), adding peripheral distractors (Subtest 3), and adding a second central object for identification with simultaneous peripheral localization in the presence of distractors (Subtest 4). For each subtest, stimulus display time was manipulated to become shorter with each trial until participants achieved a correct identification and localization on 75% of trials. Each participant received a composite score equivalent to the duration of stimulus display times (ms) corresponding to the 75% threshold for each of the 4 subtests. Stimulus display time scores were reverse-coded (higher = better) then standardized to the baseline mean (i.e., z-scored) for analyses.

##### 2.4.2.3 Everyday function

A composite variable was created from two performance-based measures assessing instrumental activities of daily living (IADL). Scores for each measure were first standardized to the baseline mean (i.e., z-scored), then summed and averaged to obtain a composite score at each timepoint. A modified version of the Observed Tasks of Daily Living test (Diehl et al., 2005; Diehl et al., 1995) objectively assesses participants’ problem-solving capabilities on everyday tasks in three domains: taking medication, using the telephone, and paying bills. Higher scores reflected better performance.

The Timed Instrumental Activities of Daily Living assessment (Owsley et al., 2001; 2002) served as a performance-based outcome measure of everyday function within the domains of telephone communication (e.g., using a phonebook to find a number), shopping (e.g., finding items on a shelf), finances (e.g., making change), medication use (e.g., reading medication directions), or nutrition (e.g., reading ingredients from a list on a can). Longer completion times reflected poorer function. For the current analyses, completion times were reverse scaled so that higher = better.

##### 2.4.2.4 Physical function

A composite variable was created from two measures assessing dynamic balance and lower body mobility. Because higher scores for each measure reflected poorer performance, scores were first reverse-coded such that higher = better, then standardized to the baseline mean (i.e., z-scored), summed, and averaged to obtain a composite score at each timepoint. The Turn 360 test (Berg et al., 1992; Dite and Temple, 2002) served as a performance-based measure of dynamic balance. While standing in place, participants completed a full 360-degree turns as quickly and as safely as possible. The number of steps to complete the turn on two trials were averaged to obtain the Turn 360 score.

The Timed Get Up and Go (TUG) test (Podsiadlo and Richardson, 1991) was used to assess lower body mobility. The total time taken to stand up from a seated position, walk in a straight line for 3 m, turn around, walk back, and return to the same seated position on two separate trials were averaged to obtain a TUG score.

### 2.5 Analysis plan

#### 2.5.1 Assessing feasibility

Feasibility indicators included the length of time required to complete participant recruitment, the proportion of screened individuals who were subsequently randomized (i.e., enrollment rate), intervention adherence and retention rates, and missing assessment data. Additionally, reasons for dropout were noted. The immediate post-test retention rate was calculated as the proportion of randomized participants who remained enrolled at the immediate post-test assessment visit. The 6-month follow-up retention rate was based on the subset of randomized participants who were invited to return for the 6-month follow-up visit (n = 40). Missing data included any missing outcome assessments regardless of enrollment status.

#### 2.5.2 Estimating variability in functional changes at each immediate post-test and 6-month follow-up

Difference scores were calculated for executive, UFOV, everyday, and physical function by subtracting the baseline score from: 1) immediate post-test score, and 2) 6-month follow-up score. The mean, standard deviation (SD), and 95% confidence intervals (CIs) in change scores were computed for the total sample and by study arm for each functional outcome immediately post-intervention and at 6-month follow-up. Statistical analyses were conducted using SPSS 27.0.

#### 2.5.3 Visualizing patterns of functional performance over time by study arm

Prior to generating graphs, each of the functional outcome scores was first standardized relative to the baseline mean. Functional outcome scores were standardized relative to the baseline mean of the total sample (N = 55) for baseline to immediate post-test graphs. Because only a subset of participants (n = 40) was invited back for 6-month follow-up, functional outcomes scores for baseline to 6-month follow-up graphs were standardized relative to the baseline mean of the follow-up subsample.

## 3 Results

Table 1 displays the baseline characteristics of the enrolled sample (N = 55). Enrolled participants ranged in age from 65 to 85 years (M = 71.4, SD = 5.3). Most of the sample were white (87%), male (55%), and had a bachelor’s degree or higher (73%).

**TABLE 1:** Baseline participant characteristics, number of training sessions completed, and missing assessment visits.

Variable	Total (N = 55)	Cognitive (n = 15)	Exercise (n = 13)	Exergame (n = 14)	Control (n = 13)
	Mean (SD)	Mean (SD)	Mean (SD)	Mean (SD)	Mean (SD)
Age	71.4 (5.3)	68.6 (3.3)	73.3 (5.8)	73.6 (6.2)	70.5 (4.0)
Female (%)	45.5	46.7	46.2	42.9	46.2
Non-White (%)	12.7	20.0	7.7	14.3	7.7
Education (years)	16.0 (2.4)	16.3 (0.5)	15.5 (0.5)	16.1 (0.6)	16.2 (0.5)
Executive Function	0.03 (0.7)	0.05 (0.7)	0.03 (0.8)	−0.24 (0.7)	0.29 (0.8)
Useful Field of View Function	−831.5 (287.8)	−738.7 (277.2)	−921.7 (300.5)	−897.8 (262.8)	−777.1 (300.1)
Everyday Function	0.02 (0.7)	0.02 (0.6)	0.11 (0.6)	−0.08 (0.8)	0.03 (0.7)
Physical Function	0.00 (0.9)	0.01 (0.8)	0.09 (1.0)	0.13 (1.0)	−0.24 (0.7)
Training Sessions Completed	19.0 (3.2)	18.9 (3.9)	20.0 (0.0)	18.1 (3.6)	N/A
Missing Immediate Post-Test Assessment	7/55	1/15	1/13	3/14	2/13
Missing 6-Month Follow-Up Assessment	0/40	0/10	0/9	0/9	0/12

Note: *Differed from Exercise (*p* = .02) and Exergame (*p* = .01) arms.

### 3.1 Assessing feasibility

#### 3.1.1 Recruitment, enrollment and intervention adherence

The time to complete recruitment was 13 months. During that time, 208 individuals were screened for the study. Of those, 87 did not meet eligibility requirements, 51 did not return the physical consent packet, and 12 either declined or did not participate for other reasons (see Figure 1 for Consort diagram). The resulting enrollment rate was 26% (i.e., proportion of screened to randomized individuals). Table 1 shows training adherence rates and number of missing assessments by study arm. Of the 42 participants assigned to intervention training, 35 (81%) completed all 20 training sessions. The median number of training sessions completed in each of the three intervention training groups was 20.

#### 3.1.2 Retention and missing data

Six participants withdrew from the study after being randomized to COG (n = 1), EX (n = 1), EXCOG (n = 3), and CON (n = 1). This equated to an overall retention rate of 89% and retention by study arm ranging from 79% (EXCOG) to 93% (COG) at immediate post-test. All participants invited back for a 6-month follow-up were retained (n = 40). Among those assigned to intervention training, one participant completed five COG training sessions prior to dropout. Three participants randomized to EXCOG completed between 10 and 14 training sessions prior to withdrawing. No study withdrawals were due to intervention-related adverse events.

Participants who dropped out were not statistically different than study completers with regard to age, education, race, gender, or baseline functional outcome measures (*p* > .05). Likewise, participants who completed a 6-month follow-up were not statistically different from those who completed only the post-test visits in the above characteristics (*p* > .05). Among retained participants, immediate post-test data were missing for one CON arm participant who completed a subsequent 6-month follow-up assessment.

### 3.2 Variability estimates for functional changes at immediate post-test and 6-month follow-up

Mean change scores with 95% CIs for executive function, UFOV function, physical function, and everyday function are reported by study arm and for the total sample in Table 2 (baseline to immediate post-test) and Table 3 (baseline to 6-month follow-up). Change scores for functional outcomes from baseline to immediate post-test were mostly negative across study arms. Exceptions were a small positive change in executive function for EXCOG, positive change in UFOV function for all study arms, a positive change in physical function for EXCOG, and a positive change in everyday function for COG. Among the positive change scores for UFOV function, 95% CIs for the CON arm contained zero but the three training arms did not. The 95% CIs for negative changes in executive function and everyday function for CON arm did not contain zero. Otherwise, all 95% CIs for baseline to immediate post-test changes included zero. Mean change scores followed similar patterns from baseline to 6-month follow-up.

**TABLE 2:** Changes in functional outcomes from baseline to immediate post-test.

Functional outcome	Study arm	n	Mean	SD	95% confidence interval		Minimum	Maximum
					Lower	Upper		
Executive	Cognitive	14	−0.11	0.25	−0.25	0.04	−0.60	0.31
Function	Exercise	12	−0.01	0.38	−0.26	0.23	−0.57	0.67
	Exergame	11	0.05	0.36	−0.19	0.29	−0.26	0.84
	Control	11	−0.30	0.32	−0.52	−0.08	−0.92	0.17
	Total	48	−0.09	0.34	−0.19	0.01	−0.92	0.84
UFOV Function	Cognitive	14	273.50	214.37	149.72	397.28	−126.00	737.00
	Exercise	12	155.67	188.96	35.61	275.72	−169.00	464.00
	Exergame	11	152.91	138.91	59.59	246.23	−21.00	380.00
	Control	11	46.91	193.81	−83.30	177.11	−154.00	492.00
	Total	48	164.48	199.98	106.41	222.55	−169.00	737.00
Physical	Cognitive	14	−0.01	0.66	−0.40	0.37	−1.09	0.89
Function	Exercise	12	−0.26	0.69	−0.70	0.18	−1.29	0.79
	Exergame	11	0.21	0.98	−0.45	0.87	−1.54	1.20
	Control	11	−0.07	0.56	−0.44	0.31	−1.25	0.53
	Total	48	−0.04	0.73	−0.25	0.18	−1.54	1.20
Everyday	Cognitive	14	0.09	0.48	−0.18	0.37	−0.67	0.94
Function	Exercise	12	−0.09	0.54	−0.43	0.26	−1.03	0.88
	Exergame	11	−0.02	0.54	−0.39	0.34	−0.91	0.85
	Control	11	−0.21	0.27	−0.39	−0.03	−0.49	0.30
	Total	48	−0.05	0.47	−0.18	0.09	−1.03	0.94

Note: UFOV, Useful Field of View. Higher scores reflect better performance for all functional outcomes.

**TABLE 3:** Changes in functional outcomes from baseline to 6-month follow-up.

Functional outcome	Study arm	n	Mean	SD	95% confidence interval		Minimum	Maximum
					Lower	Upper		
Executive	Cognitive	10	−0.03	0.22	−0.19	0.12	−0.53	0.19
Function	Exercise	9	−0.01	0.39	−0.31	0.29	−0.55	0.57
	Exergame	9	0.22	0.43	−0.11	0.56	−0.39	0.96
	Control	12	−0.22	0.35	−0.44	0.00	−0.70	0.37
	Total	40	−0.03	0.38	−0.15	0.09	−0.70	0.96
UFOV	Cognitive	10	217.00	193.78	78.38	355.62	−49.00	549.00
Function	Exercise	9	272.00	122.81	177.60	366.40	140.00	547.00
	Exergame	9	253.56	172.61	120.88	386.24	−17.00	550.00
	Control	12	102.67	195.09	−21.29	226.62	−241.00	389.00
	Total	40	203.30	182.87	144.81	261.79	−241.00	550.00
Physical	Cognitive	10	0.07	0.53	−0.31	0.45	−0.54	1.16
Function	Exercise	9	0.05	0.73	−0.52	0.61	−0.88	0.95
	Exergame	9	0.18	0.68	−0.34	0.70	−1.09	1.17
	Control	12	−0.06	0.63	−0.46	0.34	−1.09	0.70
	Total	40	0.05	0.62	−0.15	0.25	−1.09	1.17
Everyday	Cognitive	10	0.23	0.36	−0.03	0.50	−0.23	0.99
Function	Exercise	9	−0.02	0.53	−0.42	0.39	−0.69	0.9
	Exergame	9	−0.02	0.53	−0.42	0.39	−0.75	0.73
	Control	12	−0.05	0.45	−0.34	0.23	−0.89	0.55
	Total	40	0.04	0.46	−0.11	0.18	−0.89	0.99

Note: UFOV, Useful Field of View. Higher scores reflect better performance for all functional outcomes.

### 3.3 Patterns of functional performance over time

**FIGURE 2:**
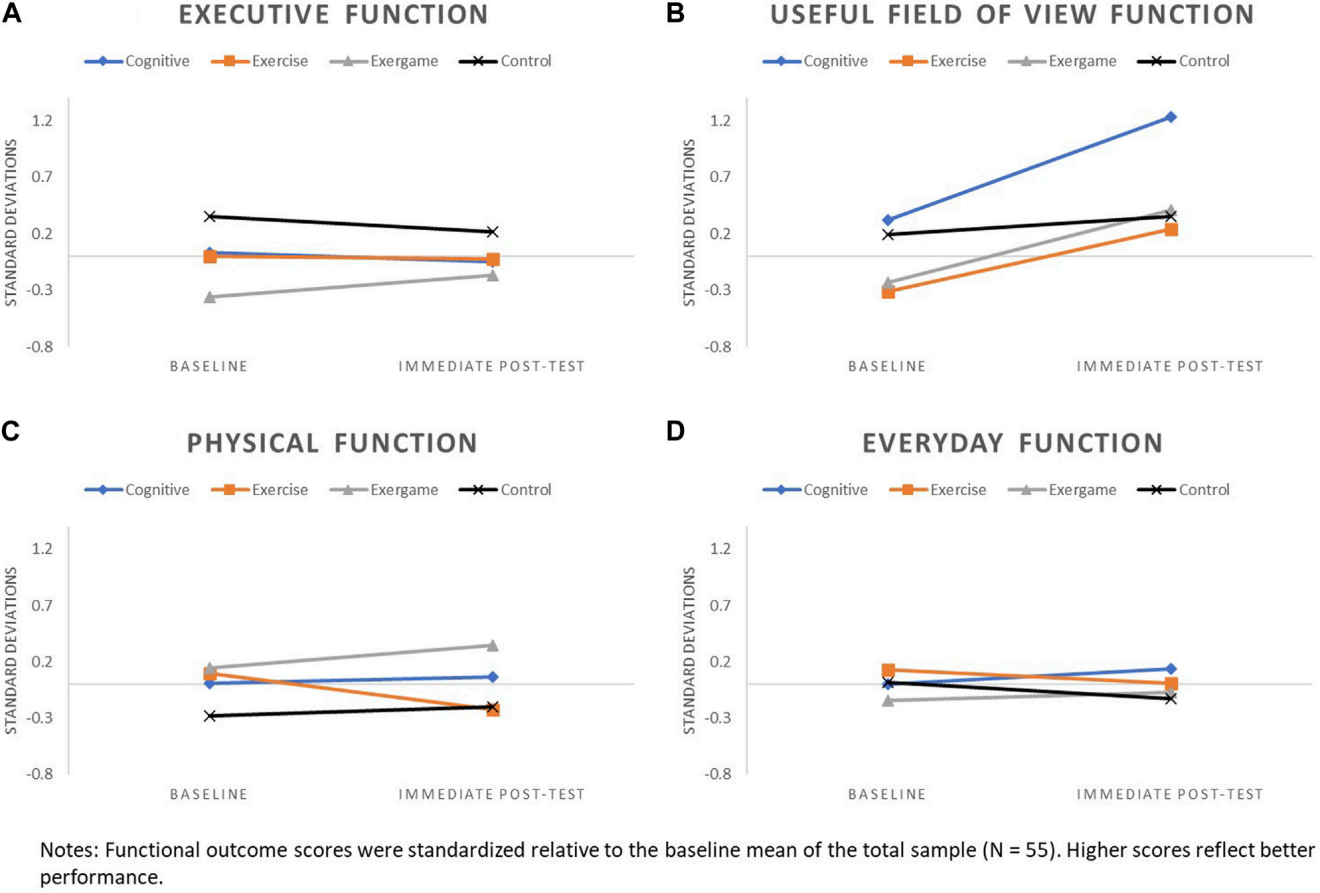
Patterns of change from baseline to immediate post-test for: **(A)** Executive Function, **(B)** UFOV Function, **(C)** Physical Function, and **(D)** Everyday function.

**FIGURE 3:**
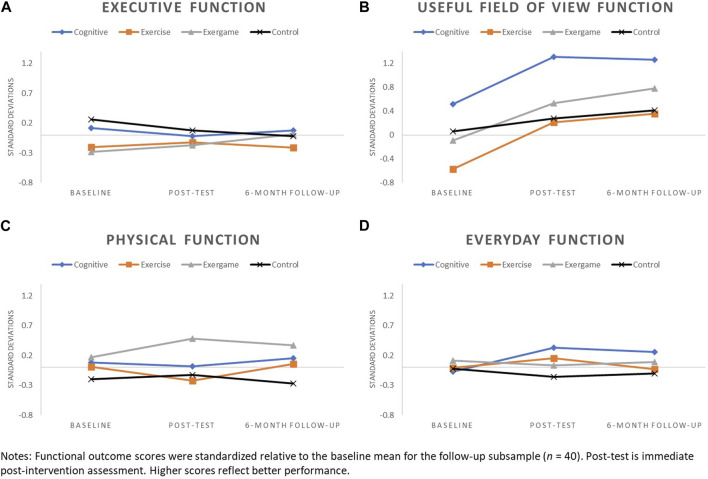
Patterns of change from baseline to 6-month follow-up for: **(A)** Executive Function, **(B)** UFOV Function, **(C)** Physical Function, and **(D)** Everyday function.

Patterns of change for each of the four functional outcomes by study arm are displayed in Figure 2 (baseline to immediate post-test) and Figure 3 (baseline to 6-month follow-up).

#### 3.3.1 Executive function

Patterns of change for executive function were generally flat or slightly declined for COG, EX, and CON arms across assessment points, with more pronounced decline observed for CON, relative to other arms, at the 6-month follow-up. Executive function improved from baseline to immediate post-test for the EXCOG arm and continued an upward trajectory from immediate post-test to 6-month follow-up.

#### 3.3.2 UFOV function

Changes in UFOV function from baseline to immediate post-test were positive for all study arms. All training arms had more pronounced improvements in UFOV relative to CON, with the greatest improvement observed for COG. Less pronounced upward trajectories in UFOV function continued from immediate post-test to 6-month follow-up for EXCOG, EX and CON arms, while COG had a small decline.

#### 3.3.3 Physical function

Little to no change in physical function was observed for COG and CON arms across assessment points. Physical function improved for the EXCOG arm from baseline to immediate post-test and these improvements appeared to persist with only a small decline at 6-month follow-up. The EX arm declined in physical function from baseline to immediate post-test, but showed a return to baseline levels at 6-month follow-up.

#### 3.3.4 Everyday function

Subtle negative to no changes in everyday function were observed for EX, EXCOG and CON arms across assessment points. Small improvements in everyday function were found for the COG arm from baseline to immediate post-test, with only a slight decline from immediate post-test to 6-month follow-up.

## 4 Discussion

A great deal of research has demonstrated interventions can help preserve or improve older adults’ functional abilities (e.g., Ball et al., 2007). However, many previous studies compared experiment-specific interventions (Dajime et al., 2021) or limited comparisons to one or two intervention types (e.g., Chen et al., 2021). There is limited understanding about the effectiveness of different commercially available interventions and how they may differentially impact functional outcomes across performance domains. The goals of this preliminary study were to establish the feasibility of conducting a trial directly comparing three distinct intervention types, provide estimates of variability in functional outcomes, and explore patterns of change associated with each of these commercially available interventions.

First, results generally support the feasibility of conducting a larger scale trial employing most of the same general procedures. Among the feasibility indicators, intervention adherence was particularly high, with 95% of assigned training sessions completed across study arms and 81% of training arm participants completing all 20 of the required sessions. Retention rates were also high overall (89%) and across study arms-- ranging from 79% to 93%. High adherence and retention rates were likely aided by the decision to deliver the intervention in dyads (Carr et al., 2019), which provided built-in social support, encouragement, and peer accountability. This may have also contributed to nearly even numbers of men and women participants, as spousal couples were allowed to enroll and undergo randomization together. Other probable contributors to high adherence were the relatively short 10-week intervention duration and twice weekly training session frequency, given that longer intervention durations and infrequent (one weekly session) have been associated with lower adherence to physical exercise in older adults (ColladoMateo et al., 2021). Additionally, it is unclear to what extent, if any, receiving financial rewards for completing training motivated training adherence. Although there is mixed evidence supporting non-goal-contingent, delayed financial reinforcers, such as those provided in the current study, for motivating health behaviors (Tambor et al., 2016; Adams et al., 2022), it is possible they played a role in encouraging training behaviors and completing assessment visits. An avenue for future research is to test different reward mechanisms/schedules for intervention adherence and long-term health behavior adoption and maintenance among older adults.

The screening/enrollment process implemented in this pilot trial yielded 208 screened participants in a 13-month period and a screening-to-randomization rate of 26%. This is potentially problematic for scaling up to a large-scale effectiveness trial. For example, assuming even a conservative increase to 75 randomized participants per arm, this would translate to 1,135 screenings needed to randomize 300 participants. We recommend reevaluating eligibility criteria for a large-scale trial, to increase the likelihood that individuals who meet eligibility criteria at telephone screening will subsequently be randomized. Specifically, we propose modifying the requirement that initially eligible candidates obtain written permission from their health provider before attending a baseline assessment visit. This requirement was largely precautionary; the physical activities in our study were not intensive. Approximately 25% of the 208 screened individuals that met initial eligibility criteria did not return their physician packet, and as a result, did not progress to the baseline assessment visit. This barrier potentially excluded participants who would otherwise be eligible for participation. Other possible modifications for a large-scale trial are to recruit directly through health providers to facilitate consent, and/or to incorporate a clinical physical assessment at the baseline visit to eliminate the need for physician/health provider consent for low-risk individuals.

We found the most notable functional changes and differences in patterns of change across study arms for UFOV function. We observed improvements equating to 0.91, 0.64, 0.55, and 0.16 standard deviations (SD) from baseline to immediate post-test for the cognitive training, exergame, exercise, and control arms, respectively. Patterns mostly persisted at 6-month follow-up, suggesting that intervention-related improvements in UFOV function may be somewhat durable. The large and lasting improvements found for the cognitive training arm were not surprising because the training tasks were similar to the UFOV subtests that tap into individuals’ visual attention and visual speed of processing abilities (Edwards et al., 2015). Previous research has consistently demonstrated the short-term and maintained effects of this kind of training on UFOV function (Edwards et al., 2005; Ball et al., 2007; Belchior et al., 2019). UFOV improvements found for the exercise and exergame training arms and to a lesser extent, the control arm, suggest that UFOV tasks may be sensitive to practice effects and/or the effects of multiple training interventions, or may have been impacted by some other variable not accounted for in our descriptive analyses.

Changes in executive, physical and everyday function from baseline to immediate post-test and to 6-month follow-up, if any, were subtle and mostly negative regardless of study arm. Exceptions were baseline to immediate post-test improvements of 0.19 SD in executive function and 0.20 SD in physical function for the exergame training arm, and an improvement of 0.14 SD in everyday function for the cognitive training arm. Although this pilot study was not powered to test intervention effectiveness, we expected to see patterns of executive and physical function improvements for the exercise training arm based on prior research (Erickson et al., 2011; Muiños and Ballesteros, 2018; Dipietro et al., 2019). It is especially surprising that mean physical function declined by 0.32 SD from baseline to immediate post-test in this group and then returned to nearly baseline by the 6-month follow-up. Because we saw no extreme outliers in the data that may have driven these unexpected patterns of results, we offer several conceivable study and training design factors that may have been contributing factors.

The first, and perhaps most important, possible explanation for unexpected patterns of results for the exercise training arm was that exercise dose provided in our training was too low. Specifically, our exercise intervention fell short of the 150 min of weekly recommended amount of moderate-intensity aerobic activity (American College of Sports Medicine et al., 2009) and did not follow principles of progression for resistance training recommended to promote physical function improvements (American College of Sports Medicine, 2009). As their names suggest, each of the videos targeted different age groups and/or functional abilities (i.e., Fit at Any Age, Older and Wiser, and Older and Much Wiser). However, all participants regardless of age or abilities participated in all three videos presented in a random order. Thus, the intervention may not have been optimized to promote physical functioning improvements for all participants with regard to intensity, progressive overload, or overall volume. A second potential explanation for the unexpected patterns of physical function change for the exercise training arm was a mismatch between our physical function outcome measure, consisting of two reliable and valid lower body physical function measures, and the domains of physical function that may have benefitted from the exercise training. To ensure a larger scale efficacy trial is not adversely impacted by these two potential design flaws, we recommend the following: 1) Ensure the exercise training intervention meets dosing recommendations set forth by the American College of Sports Medicine, incorporates methods for tailoring dosages to individual abilities and fitness levels, and integrates principles of progressive overload, and 2) Implement a comprehensive physical assessment battery to include more measures across multiple dimensions of physical functioning to maximize the likelihood of detecting intervention effects.

### 4.1 Strengths and limitations

This pilot randomized controlled trial had several notable strengths. First, we used three different commercially available behavioral interventions, which if deemed successful at improving functional outcomes, can provide direct translation. Second, outcomes included cognitive, physical, and everyday functional abilities, all of which are relevant to older adult wellbeing and independence. Perhaps most consequentially, our study illustrates the feasibility of conducting a fully powered randomized controlled trial employing the same procedures. Notably, the selected interventions were economical and had high adherence rates among enrolled participants, with 81% completing all the 20 required training sessions. Additionally, the decision to randomize in dyads for training likely aided adherence by building in peer social support, encouragement, and accountability.

However, the study is not without limitations. First, it is possible that expectation or placebo effects contributed to the large changes in UFOV function for the cognitive training arm. Though participants were not given any detail about the specific benefits of their training, only that different brain and exercise programs “designed to improve brain and physical health” were being investigated, it was likely they recognized their training as a brain program vs an exercise program.

Following recommendations provided in Masurovsky’s systematic review (2020), this limitation can be addressed in a larger scale trial by incorporating stronger placebo control methodology, including a similar-form active control group and assessing participant expectations before and after training.

The study also included a relatively homogenous group of self-selected participants A larger scale trial should aim to recruit a more diverse—in race/ethnicity, health status, and education level—sample to generalize results to a more representative aging population. As we already noted, the added step of getting a doctor’s signed consent resulted in a low screening-to-randomization rate. It is also possible that this added step may have 1) limited participation from those who were unable to contact their doctor in the required time period, 2) excluded those who did not have a doctor, or 3) excluded those without a high enough level of motivation to complete the additional steps to needed enroll (e.g., some doctor’s may have required an in-office visit and/or additional medical tests to provide consent). We believe that modifying or eliminating the physician’s consent requirement for a larger scale trial may not only enable more efficient recruitment but will also increase the likelihood of enrolling a more diverse sample. It may also lead to the negative outcome of enrolling participants with lower motivation or with more barriers to adherence who would be less likely to complete study visits or training once enrolled.

## 5 Conclusion

The results of this pilot study lend support to conducting a fully powered randomized trial testing and directly comparing the effects of commercially available cognitive, physical, and combined exergame interventions on multiple facets of functioning in older adults. Several recommended modifications to the pilot study procedures include modifying or removing the requirement for physician’s consent for participation, reevaluating the exercise training intervention to ensure American College of Sports Medicine recommendations for dosage are met, and including a more comprehensive physical function battery.

## Data Availability

The raw data supporting the conclusion of this article will be made available by the authors, without undue reservation.
